# Use of Digital Tools in Arbovirus Surveillance: Scoping Review

**DOI:** 10.2196/57476

**Published:** 2024-11-18

**Authors:** Carolina Lopes Melo, Larissa Rangel Mageste, Lusiele Guaraldo, Daniela Polessa Paula, Mayumi Duarte Wakimoto

**Affiliations:** 1 Instituto Nacional de Infectologia Evandro Chagas Fundação Oswaldo Cruz Rio de Janeiro Brazil; 2 Instituto Brasileiro de Geografia e Estatística Rio de Janeiro Brazil

**Keywords:** arbovirus infections, dengue, zika virus, chikungunya fever, public health surveillance, digital tool, technology

## Abstract

**Background:**

The development of technology and information systems has led to important changes in public health surveillance.

**Objective:**

This scoping review aimed to assess the available evidence and gather information about the use of digital tools for arbovirus (dengue virus [DENV], zika virus [ZIKV], and chikungunya virus [CHIKV]) surveillance.

**Methods:**

The databases used were MEDLINE, SCIELO, LILACS, SCOPUS, Web of Science, and EMBASE. The inclusion criterion was defined as studies that described the use of digital tools in arbovirus surveillance. The exclusion criteria were defined as follows: letters, editorials, reviews, case reports, series of cases, descriptive epidemiological studies, laboratory and vaccine studies, economic evaluation studies, and studies that did not clearly describe the use of digital tools in surveillance. Results were evaluated in the following steps: monitoring of outbreaks or epidemics, tracking of cases, identification of rumors, decision-making by health agencies, communication (cases and bulletins), and dissemination of information to society).

**Results:**

Of the 2227 studies retrieved based on screening by title, abstract, and full-text reading, 68 (3%) studies were included. The most frequent digital tools used in arbovirus surveillance were apps (n=24, 35%) and Twitter, currently called X (n=22, 32%). These were mostly used to support the traditional surveillance system, strengthening aspects such as information timeliness, acceptability, flexibility, monitoring of outbreaks or epidemics, detection and tracking of cases, and simplicity. The use of apps to disseminate information to society (*P*=.02), communicate (cases and bulletins; *P*=.01), and simplicity (*P*=.03) and the use of Twitter to identify rumors (*P*=.008) were statistically relevant in evaluating scores. This scoping review had some limitations related to the choice of DENV, ZIKV, and CHIKV as arboviruses, due to their clinical and epidemiological importance.

**Conclusions:**

In the contemporary scenario, it is no longer possible to ignore the use of web data or social media as a complementary strategy to health surveillance. However, it is important that efforts be combined to develop new methods that can ensure the quality of information and the adoption of systematic measures to maintain the integrity and reliability of digital tools’ data, considering ethical aspects.

## Introduction

Arboviruses have become relevant public health problems in tropical and subtropical areas due to either socioeconomic or environmental factors, involving inadequate occupation of urban space, poor sanitary conditions, and deforestation [1].

Dengue, caused by the dengue virus (DENV), is 1 of the most important neglected tropical diseases transmitted by mosquitoes in humans. Since its onset in Southeast Asia in the 1950s, the disease has rapidly spread throughout tropical regions and currently remains a health concern worldwide [2].

Chikungunya, caused by the chikungunya virus (CHIKV), was first described in 1952 [3] and has been responsible for outbreaks and epidemics in Asia and Africa. Between 2005 and 2007, 266,000 cases were reported in the Reunions Islands, affecting almost 34% of the island’s population [4]. A major outbreak occurred in 2013 with the emergence of an Asian lineage of the virus affecting the Caribbean Saint Martin Island, from where the virus spread to more than 50 countries of the South American continent, leading to over 1 million infections [5]. It is important to highlight the chikungunya disease burden, which includes chronicity, severe infections, increased hospitalization risks, and associated mortality [6].

Zika was first described in 1947 in nonhuman primates, and infections in humans were sporadic and mild. In 2013-2014, an outbreak occurred in French Polynesia, and severe neurological manifestations were reported [7]. In 2015, cases of congenital microcephaly among pregnant women infected with zika virus (ZIKV) were reported in Brazil. Those findings raised concerns about the infection during pregnancy, and a body of evidence showed the association of ZIKV infection with fetal death, growth restriction, and a series of abnormalities in the fetal central nervous system, as well as microcephaly [8,9].

The infection scenario caused by arboviruses has pointed to relevant threats to public health in recent years. The cocirculation of the 3 arboviruses (DENV, CHIKV, ZIKV) has imposed major challenges in surveillance and increased the demand for health service support in affected areas [10]. Among the arboviruses, we chose DENV, CHIKV, and ZIKV as they have great medical importance and similar clinical manifestations and as transmission occurs through the same vector, the mosquito *Aedes aegypti*. Dengue fever has a high incidence in several countries. Chikungunya presents high morbidity, considering the course of the disease in the acute phase, which can lead to chronic symptoms. Zika in pregnant women has an important impact on congenital neurological manifestations, in addition to being an emerging disease.

The continuous and systematic collection, analysis, and interpretation of health-related data are part of the scope of public health surveillance. Monitoring of outbreaks and epidemics, tracking of cases, evaluation of interventions, evaluation of rumors, communication (cases and bulletins), and decision-making by health agencies are essential steps for a surveillance system. Therefore, the effectiveness of a surveillance system is directly related to its ability to control diseases [11,12]. However, the rapid development of data science, including big data and artificial intelligence (AI), and the growth of accessible and heterogeneous health-related data are definitely changing the field of health surveillance [13]. The use of technology in health care has increased in recent years. Digital tools, such as apps, digital forms, online chats, video calls, telemedicine, social media, and games, have been used for data collection, case tracking, and disease risk classification [14-16]. Furthermore, the use of big data composing hybrid systems, through the combination of structured and unstructured data, is a promising strategy to collect electronic health records in real time, potentially impacting infectious disease surveillance [17].

It is noteworthy that public engagement in the digital technological universe is becoming increasingly important worldwide. Therefore, the use of digital tools for arbovirus surveillance seems relevant due to the impact of the diseases and the need for timely and effective control strategies [18].

This review aimed to assess the available evidence and gather information about the use of digital tools for arbovirus (DENV, ZIKV, and CHIKV) surveillance.

## Methods

### Study Design

We conducted a scoping review to systematically map the use of digital tools in arbovirus surveillance [19], defined as technological resources/electronic devices capable of establishing communication between individuals through data sharing [20,21]. Our protocol was elaborated using the Joanna Briggs Institute PRIMSA-ScR (Preferred Reporting Items for Systematic Reviews and Meta-Analyses Extension for Scoping Reviews) guidelines for scoping reviews (Table S1 in Multimedia Appendix 1) [19,22]. The protocol was registered with the Open Science Framework.

### Search

Data were retrieved from the following bibliographic databases: MEDLINE, EMBASE, LILACS, SCIELO, Web of Science, and SCOPUS. The research question was based on the population, context, concept (PCC) approach [19]: population, digital tools; context, arbovirus surveillance; and concept, use of digital tools to perform surveillance. The following Medical Subject Headings (MeSH) descriptors were combined: “arbovirus infections,” “dengue,” “zika virus,” “chikungunya fever,” “public health surveillance,” “epidemiological monitoring,” “technology,” “audiovisual aids,” “social media,” “big data,” “mobile apps,” and “social networking.” The search was performed in April 2023 (Table S2 in Multimedia Appendix 2).

### Selection, Reading, and Data Extraction

The selection was independently performed by 2 authors (CLM and LR), and disagreements were resolved by a third author (MDW). The inclusion criterion was defined as studies that described the use of digital tools in arbovirus surveillance.

First, titles and abstracts returned by the search were read, and the following were excluded: letters, editorials, reviews, case reports, series of cases, descriptive epidemiological studies, laboratory and vaccine studies, economic evaluation studies, and studies that did not clearly describe the use of digital tools in surveillance. Next, potentially eligible studies were read in full, and the same inclusion and exclusion criteria were applied. The online software Rayyan was used for the selection process [23].

Data from included studies were collected using a standardized data extraction tool designed for this study. The form included the following sections: identification of the study (authors, journal, year of publication, language), studies characteristics (period, study population, place of study), digital tools used, frequency of data collection, objective of the method used for the surveillance of arboviruses, and practical applicability. Data charting was implemented using EpiData 3.1 software.

The form was tested initially with 5 papers and subsequently subjected to minor adjustments, such as including new data record fields or changes in format to improve information recording. Two reviewers independently collected data from each included paper. Any disagreements were resolved through discussion between the 2 reviewers or by a third reviewer.

### Data Synthesis and Analysis

A descriptive analysis of the methods and results of using digital tools was carried out based on the attributes for evaluating surveillance systems proposed by the Centers for Disease Control and Prevention (CDC) and on some essential surveillance activities. We used the following attributes: (1) sensitivity (proportion and cases of the disease detected by the surveillance system, ability to detect outbreaks, ability to monitor changes in the number of cases over time), (2) opportunity (speed between the steps of the surveillance system), (3) simplicity (structure and ease of operationalization of the surveillance system), (4) acceptability (willingness of people or organizations to participate in the system), (5) flexibility (ability to adapt to changing information needs and operating conditions with minimal need for time, personnel, and resources), (6) specificity (capacity of the system to exclude “noncases” of the disease), and (7) positive predictive value (PPV: proportion of reported cases that actually have the event under surveillance) [24]. Furthermore, the criteria for evaluating the contributions of digital tools to arbovirus surveillance were applied based on the *Epidemiological Surveillance Guide*, and the International Health Regulations (IHR) [25,26]. The following surveillance activities were used: monitoring of outbreaks or epidemics, tracking of cases, decision-making by health agencies, identification of rumors, communication (cases and bulletins), and dissemination of information to society.

If the attributes or activities were not clearly mentioned in the study, each independent observer (CLM and LR) imputed the presence or absence according to the CDC definition or according to the performance of the surveillance system. A third observer (MDW) resolved conflicts. A dichotomous variable was created to evaluate the presence or absence of the contribution of digital tools to arbovirus surveillance. An arbitrary value of 2 was assigned when the attributes sensitivity, opportunity, simplicity, acceptability, flexibility, specificity, and PPV were present and 1 if they were absent.

The sum of the scores of the 7 attributes was calculated for each of the selected studies, and this sum variable was categorized as follows: 7 or 8, unsatisfactory; 9 or 10, moderately satisfactory; 11 or 12, satisfactory; and 13 or 14, very satisfactory. The sum of the scores of the 6 health surveillance activities (monitoring of outbreaks or epidemics, tracking of cases, decision-making by health agencies, identification of rumors, communication [cases and bulletins], and dissemination of information to society) was performed for each of the selected studies, and this sum variable was categorized as follows: 6, unsatisfactory; 7 or 8, moderately satisfactory; 9 or 10, satisfactory; and 11 or 12, very satisfactory.

Next, the percentage of the use of each digital tool in the reviewed studies was calculated. The frequency with which the 7 health surveillance attributes and 6 activities were present in the included studies was also calculated according to the use of digital tools (Multimedia Appendices 3 and 4).

The mean (SD) and *P* value of the sum attributed to the score of each axis were calculated: 7 surveillance system attributes and 6 health surveillance activities according to the digital tools that were used with the greatest value absolute in the included studies (apps, Twitter, Google Trends, and big data), with the aim of identifying whether the groups had a statistically significant difference. Subsequently, we compared the means using ANOVA for the aforementioned digital tools according to the 2 axes to verify the difference between them.

We used R software version 4.3.1 (*summarytools* package for descriptive analysis, *dplyr* package for working with dataframes, and *MASS* and *car* native R packages for ANOVA; R Foundation for Statistical Computing) to perform data analysis.

## Results

### Study Characteristics

The search strategy retrieved 2227 studies; after removing duplicates and applying the inclusion and exclusion criteria, we included 68 (3%) studies in the review (Figure S1 in Multimedia Appendix 1).

The characteristics of the studies included are outlined in Multimedia Appendix 5. Of the 68 studies, 50% (n=34) were performed in Asia, 16% (n=11) in North America, and 9% (n=6) in Europe or Central America. In addition, 32% (n=22) of the studies were performed in South America, with 17 (77%) of these in Brazil. Dengue was addressed in 50 (74%) studies, followed by zika (n=21, 31%) and chikungunya (n=9, 13%); 6 (9%) studies addressed the 3 arboviruses simultaneously.

The digital tools most studied were apps (n=24, 35%); Twitter, currently called X (n=22, 32%); Google Trends (n=7, 10%); and big data (n=6, 9%). Social media (Twitter, Facebook, Instagram, YouTube, Flirck, Sina Weibo, and blogs) was used in 37% (n=25) of the studies.

The time range between health data collection and dissemination of information was defined by the studies as real time (n=48, 71%), near real time (n=2, 3%), yearly (n=1, 2%), weekly (n=7, 10%), daily (n=8, 12%), other (n=1, 2%), and not clearly described (n=9, 13%), as shown in Multimedia Appendix 5.

Data from digital tools were compared with official data in 16 (24%) studies: [27-42]. Of these, 12 (75%) studies [27,29,31,33-40,42] showed statistically significant correlations (*P*<.05) or strong correlations between official and unofficial data coming from online trends, social media, or big data, of which 6 (50%) addressed the use of Twitter data.

In addition, 21 (31%) studies [28,29,32,34,35,43-58] developed predictions, forecasts, detection of reemergent events, and early warning models, while 7 (10%) studies [44,59-64] presented results regarding the use of digital tools to design a participatory syndromic surveillance system, and 32 (47%) studies evaluated the use of digital tools for several surveillance activities: prevention and control of arboviruses [40,51,59,65-82], content analysis and rumors [83-91], and mitigation of the lack of epidemiological data in surveillance systems [92,93].

Most studies presented an enhancement in opportunity (n=63, 93%), flexibility (n= 57, 84%), sensitivity (n=63, 93%), and simplicity (n=46, 68%) indicators. In addition, 18 (26%) studies [28,32,39,41,43,45,47,50,52,56,58-60,65,70,79,83,92] presented an enhancement in the ability of the system to exclude “noncases” of the disease (specificity) and the proportion of reported cases that actually had the event under surveillance (PPV).

Furthermore, 63 (93%) studies addressed the monitoring of outbreaks or epidemics, and 60 (88%) studies addressed case tracking. More than half of the studies improved decision-making by health agencies (n=43, 63%) and communication (cases and bulletins; n=40, 59%). Dissemination of information to society and identification of rumors were addressed in 29 (43%) studies.

**Figure 1:**
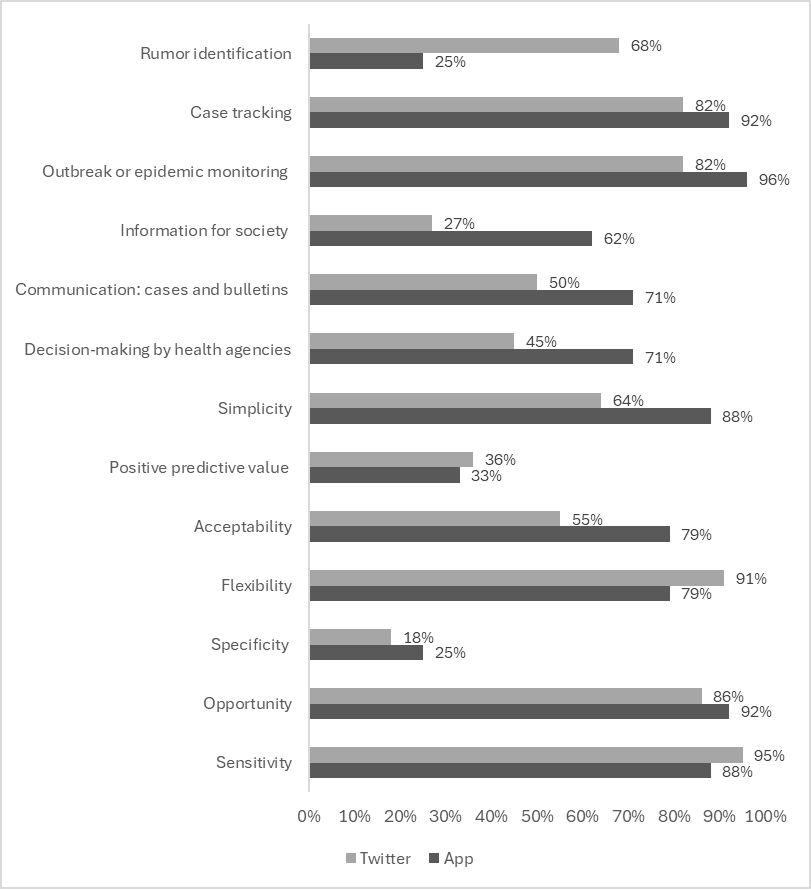
Health surveillance activities and surveillance system indicators according to the use of apps and Twitter.

The use of apps in surveillance enhanced the following indicators: opportunity (n=22, 92%), sensitivity (n=21, 88%), simplicity (n=21, 88%), acceptability (n=19, 79%), and flexibility (n=19, 79%). Furthermore, 23 (34%) studies showed that the use of apps enhanced the monitoring of outbreaks or epidemics (n=22, 96%), the tracking of cases (n=21, 92%), decision-making by health agencies (n=17, 74%), and the availability of information for society (n=14, 61%) [38,44-48,50,57,60-62,64-66,71-74,76,77,79-81,94]. Figure 1 illustrates health surveillance system attributes and contributions to health surveillance activities according to use of apps and Twitter in the 68 studies.

Of the 24 (35%) studies that mentioned the use of apps, 11 (46%) were evaluated as very satisfactory and 9 (38%) as satisfactory according to surveillance activity enhancement. In addition, 7 (29%) studies were very satisfactory and 14 (58%) were satisfactory when surveillance indicators were assessed. No study that mentioned the use of apps was evaluated as unsatisfactory.

Among the studies that addressed the use of Twitter (n=22, 32%) [27,29,30,32,35-37,41,42,49,51,63,75,82-84,86,88-92], 19 (86%) described an increase in speed between the steps of the surveillance system (opportunity) and the ability to adapt to changing information needs, while 18 (82%) described operating conditions with minimal need for time, personnel, and resources (flexibility). More than 12 (55%) studies that addressed the use of Twitter described an enhancement in system sensitivity, and 12 (55%) described the ease of operation and the willingness of people or organizations to participate in the system (acceptability). Less than 7 (32%) studies pointed out an enhancement to system specificity and the PPV.

Furthermore, 18 (82%) studies that addressed the use of Twitter described enhancement in monitoring of outbreaks or epidemics. Of these, 11 (61%) studies described an increase in communication (cases and bulletins) and decision-making by health agencies. In addition, 15 (68%) addressed the identification of rumors, while dissemination of information to society was mentioned in 6 (27%) of the studies (Figure 1).

Of the 22 studies that addressed the use of Twitter, 7 (32%) were evaluated as very satisfactory and 9 (41%) were satisfactory regarding surveillance activities. In addition, 6 (27%) studies were very satisfactory, 11 (50%) were satisfactory, 4 (18%) were moderately satisfactory, and 1 (5%) was unsatisfactory according to the analysis of surveillance system indicators.

Studies that reported the use of Google Trends (n=7, 10%) enhanced the following indicators: simplicity, sensitivity, flexibility, and opportunity. Furthermore, 6 (86%) studies enhanced the monitoring of outbreaks or epidemics and tracking of cases.

Studies that reported the use of big data (n=6, 9%) [28,30,37,43,51,93] assessed sensitivity and flexibility of the surveillance system, as well as opportunity (speed between the surveillance system steps). All of them contributed to the monitoring of outbreaks or epidemics. Additionally, 3 (50%) studies [30,43,93] addressed decision-making by health agencies.

A total of 27 (40%) studies [27,33,39-41,47,48,50,53,56, 57,61-64,67,71-73,75,79,82,85,87,91,94] were rated as very satisfactory regarding surveillance activities and addressed the use of the following digital tools: apps, Twitter, game platforms, web-based digital tools, Facebook, Google Trends, Google News, Wikipedia, and Sina Weibo. Of these, 19 (70%) investigated dengue, 7 (26%) investigated zika, and 1 (4%) addressed diarrheal syndrome, respiratory syndrome, arboviral syndrome, chikungunya, zika, and influenza.

In addition, 15 (22%) studies [27,29,36,45,50,52,59-61, 65,72,79,84,90,92] were classified as very satisfactory regarding surveillance system indicators and reported the use of the following digital tools: apps, Twitter, Google Trends, Google News, blogs, Wikipedia, telepidemiological surveillance, eletronic bracelets, Google Maps, drones, and the GPS.

In comparing the means of the sums of health surveillance system activities, there was a difference between apps, Twitter, Google Trends, and big data. The activities communication (cases and bulletins; *P*=.01), dissemination of information to society (*P*=.02), and identification of rumors (*P*=.008) showed a statistically significant difference (Table 1).

In comparing the means of the sums of health surveillance system attributes, there was a difference between apps, Twitter, Google Trends, and big data (Table 2).

However, the attribute simplicity (*P*=.03) showed a statistically significant difference.

**Table 1:** ANOVA of health surveillance activity scores.

Health surveillance system activity	Apps (n=24), mean (SD)	Big data (n=6), mean (SD)	Google Trends (n=7), mean (SD)	Twitter (n=22), mean (SD)	*P* value^a^
Decision-making by health agencies	1.7 (0.5)	1.5 (0.5)	1.4 (0.5)	1.5 (0.5)	.31
Communication: cases and bulletins	1.7 (0.5)	1.0 (0.0)	1.7 (0.5)	1.5 (0.5)	.01
Dissemination of information to society	1.6 (0.5)	1.2 (0.4)	1.1 (0.4)	1.3 (0.4)	.02
Outbreak or epidemic monitoring	2.0 (0.2)	2.0 (0.0)	1.9 (0.4)	1.8 (0.4)	.36
Rumor identification	1.2 (0.4)	1.2 (0.4)	1.3 (0.5)	1.7 (0.5)	.008
Case tracking	1.9 (0.3)	2.0 (0.0)	1.9 (0.4)	1.8 (0.4)	0.6

^a^*P* values (ANOVA) of health surveillance system activities considering the use of digital tools (apps, big data, Google Trends, and Twitter).

**Table 2:** ANOVA of health surveillance system attribute scores.

Health surveillance system attribute	Apps (n=24), mean (SD)	Big data (n=6), mean (SD)	Google Trends (n=7), mean (SD)	Twitter (n=22), mean (SD)	*P* value^a^
Sensitivity	1.9 (0.3)	2.0 (0.0)	2.0 (0.0)	1.9 (0.2)	.94
Specificity	1.8 (0.4)	1.7 (0.5)	1.2 (0.5)	1.2 (0.4)	.64
Opportunity	1.9 (0.3)	2.0 (0.0)	2.0 (0.0)	1.8 (0.4)	.88
Flexibility	1.8 (0.4)	1.7 (0.5)	2.0 (0.0)	1.9 (0.3)	.82
Acceptability	1.8 (0.4)	1.3 (0.5)	1.2 (0.4)	1.5 (0.5)	.12
PPV^b^	1.3 (0.5)	1.3 (0.5)	1.0 (0.0)	1.4 (0.5)	.67
Simplicity	1.8 (0.4)	1.0 (0.0)	1.8 (0.4)	1.5 (0.5)	.03

^a^*P* value (ANOVA) of health surveillance system attributes considering the use of digital tools (apps, big data, Google Trends, and Twitter).

^b^PPV: positive predictive value.

## Discussion

### Principal Findings

This scoping review demonstrated different approaches for the use of digital tools to prevent and control arboviruses. The use of apps and Twitter in surveillance revealed the best results.

Due to the extensive health crisis caused by COVID-19, health agencies around the world have concentrated efforts to adopt strategies aimed at providing reliable information for the population, detecting symptoms, providing first care in suspected cases, and supporting the detection of new cases and laboratory diagnosis using apps [95]. Therefore, the use of apps has proved to be beneficial, not only for surveillance, but also as a valuable aid in dealing with public health emergencies. Initiatives in Brazil, such as the COVID-19 observatory, Infogripe, and Infodengue, are used to monitor and to inform society about health problems online. In our review, 88% of the studies used a digital tool for tracking cases or monitoring arbovirus outbreaks, and most of them used social media data or apps, which is in line with the measures adopted in the response to the COVID-19 pandemic. Furthermore, our review showed a higher score for apps, indicating statistical relevance in the use of apps to disseminate information to society (*P*=.02), communication (cases and bulletins; *P*=.01), and ease of operationalization of the surveillance system (*P*=.03), with the highest means in the evaluation of scores.

Of the 24 studies that mentioned the use of apps, 96% pointed to monitoring outbreaks or epidemics and 92% mentioned tracking cases. In agreement, a systematic review conducted by Quinn et al [96] evaluated studies of web-based apps, indicator-based surveillance, and the response to communicable disease outbreaks. Their review highlighted the use of apps to improve the early detection of disease outbreaks and disease notification, as well as the active participation of users; however, they indicated a low PPV [96]. As we also observed in our review, the use of apps in arbovirus surveillance contributes either to the opportunity and ease of operationalization of the surveillance system and case detection or to a reduction in costs. Furthermore, in our review, 62% of the studies underlined the availability of information (data) in real time, which implies the triggering of timely actions with an impact on prevention and control measures. It is even more important to have real-time data in an epidemic or pandemic situation, when information and data sources need to be available in a timely manner for the implementation of infection control measures and minimization of risk factors associated with the health of the population [97].

The correlation analysis between unofficial data from social media and official data of arbovirus surveillance may be helpful to assess the potential use of nonofficial data. Twitter data on influenza was monitored for a year in the United States. Data were collected and processed based on geographic information science (GIS) and data mining and then compared with official data from national, regional, and local reports of disease outbreaks. This correlation revealed strong statistical relevance between the data sources [98]. Samaras et al [99] reported the feasibility of building an early detection and forecasting system for influenza epidemics with data from Twitter and Google search engines. This process took place in real time for 23 weeks, and the data collected from the digital tools were compared with official data. The results pointed to a high correlation with Google data and to the usefulness of Twitter data. In our review, 12 studies [27,29,31,33-40,42] showed a statistically significant correlation (*P*<.05) or strong correlation between official data of arbovirus surveillance and unofficial data from online trends, social media, or big data. Of these, more than half addressed the use of Twitter data. However, data collection and processing are crucial steps that require investment in appropriate techniques. One study showed the correlation was positive and statistically significant but with several limitations [41].

Our data showed good results regarding the use of Twitter according to either surveillance activities or surveillance system indicators. Twitter was used to propose a framework to explore online data sources to mitigate the lack of epidemiological data, assess digital behaviors and complex interaction between new data streams induced by the chikungunya outbreak, identify public health problems during a dengue epidemic, identify rumors, track and monitor cases, and support arbovirus case prediction and early warning models.

The phenomenon of misinformation and fake news became notorious during the COVID-19 pandemic, where the use of social media intensified. The sharing of fake news is a social problem that threatens public health. Jain [100] proposed an entropy approach to identify and monitor rumors related to COVID-19 based on shared tweets. In our review, the use of Twitter to identify rumors was statistically relevant in evaluating scores (*P*=.008), presenting a higher mean compared to other digital tools.

Moreover, our data showed 21 studies [28,29,32,34,35,43-58] that developed early warning models and enhanced the prediction, forecast, and detection of remergent events. The authors reported the use of apps, big data, Twitter, Google Trends, AI, the internet of things (IoT), and statistical models with algorithm adjustment to predict arbovirus cases. One study [29] presented the possibility of predicting dengue cases up to 8 weeks in advance using data from Twitter, Google Trends, and Wikipedia. In addition, the authors addressed the use of apps with geospatial and meteorological information capable of detecting and predicting possible breeding sites of the mosquito vector, predicting dengue outbreaks, generating detailed reports, and providing users with health education about dengue. Applications of fog computing were also discussed, as well as a model capable of merging large volumes of data through big data to generate early warnings. The use of Google Trends was also reported to predict the COVID-19 outbreak in India 2-3 weeks before routine surveillance [101]. Furthermore, our review showed that the use of Google Trends in arbovirus surveillance can facilitate operationalization of the surveillance system (*P*=.03), with the highest means in the evaluation of scores.

AI algorithms play a key role in rapidly predicting, detecting, classifying, sorting, and diagnosing an infection. An AI-based system is capable of accurately predicting changes in human behavior, contributing to the detection of and response to epidemic risks [102,103]. In this scoping review, one study [55] used AI and IoT as an approach to collect data and predict future situations and support preparedness and response.

Considering that we live in the big data era, and that society is increasingly connected, the use of data available on the web has been growing in several areas, although there is criticism regarding their use. The use of open, nonstructured data to obtain health outcomes requires not only the storage and processing of large volumes of data but also methodological concerns [104,105]. The balance regarding the quantity versus the quality of data remains a challenge. Nevertheless, statistical methods are being rapidly developed to meet public health demands based on the analysis of large volumes of data. The combination of data is a resource that expands the analysis capacity of a system, and this area of epidemiology is increasingly leading this scenario [105,106]. A strategy for expanding the use of social media in the surveillance area would be the “data science–based approach,” encompassing multidisciplinary teams, and “app of techniques,” with machine learning algorithms and natural language processing (NLP). In our review, the use of big data was addressed in 6 studies [28,30,37,43,51,93] that combined data from social media for tracking cases, monitoring outbreaks or epidemics, disseminating information to society, and identifying children with incomplete immunization. Moreover, the use of big data contributed to decision-making by health agencies and to prevention and control measures concerning emerging and reemerging infectious diseases.

The use of unofficial data from the internet and social media in surveillance has some limitations, such as information overload as well as uncertain quality and validity of data for surveillance purposes. Therefore, more evidence is needed regarding the efficacy and assessment of integrated systems. The phenomenon of information overload could be mitigated by investing in automated technology for monitoring health-related internet-based data so that these strategies could be adopted within the health surveillance system [105,107]. Leal et al [104] argued that in Brazil, there is an immediate lack of technological incorporation to reduce information time and improve the means used in the surveillance routine, which harms the “information for action” issue, the hallmark of public health surveillance.

Although searches by web sources are in continuous growth, it is important to emphasize that digital inclusion is limited worldwide [108]. The mitigation of biases related to the representativeness digital vehicles data is a complex process. Data about a certain disease may be underrepresented in Twitter due to the lack of digital coverage in different locations [106]. Thus, an important point to consider is the inequality of internet access to the population, which can limit the implementation of digital health surveillance strategies [109].

The validation of data through statistical techniques and other approaches is desirable to increase the reliability of the data and their use in the decision-making process for action [12]. It is important to emphasize the relevance of regulation of the use of data from digital tools to ensure the protection of the participant, especially the ethical aspects involved, even if they are available in the social networks [110].

Investment in innovation, technology, and digital tools in the routine of health surveillance is essential, especially as we are experiencing exponential technological advances and an increase in public health demands [111]. However, there is a major methodological challenge in validating information collected from unofficial sources. Additionally, it is necessary to review the regulations to support alternative and complementary surveillance systems, as described.

### Limitations

Our scoping review has some limitations related to the choice of the arboviruses DENV, ZIKV, and CHIKV, due to their clinical and epidemiological importance. Furthermore, there are some limitations regarding the assessment of accessibility and digital inclusion of the populations studied. However, these issues were not found in the included studies, despite the search including 6 databases and gray literature, without language and period restrictions.

### Conclusion

Our review outlined the use of several digital tools for arbovirus surveillance, with emphasis on the use of apps and Twitter in surveillance. These tools can contribute to surveillance in a complementary way and strengthen the following aspects: dissemination of information to society, rumor identification, information opportunity, acceptability of users to participate in the system, capacity to adapt to new epidemiological situations, monitoring of outbreaks or epidemics, case detection and tracking, operationalization of the system, and reduction in costs.

In the contemporary scenario, it is no longer possible to ignore the use of web data or social media as a complementary strategy to health surveillance. However, it is important that efforts be combined to develop new methods that can ensure the quality of information and the adoption of systematic measures to maintain the integrity and reliability of digital tools’ data, considering ethical aspects.

## Appendix

Multimedia Appendix 1 PRISMA-ScR (Preferred Reporting Items for Systematic Reviews and Meta-Analyses Extension for Scoping Reviews) checklist and PRISMA flow diagram.

Multimedia Appendix 2 Search strategy.

Multimedia Appendix 3 Health surveillance activities according to the use of digital tools.

Multimedia Appendix 4 Health surveillance system attributes according to the use of digital tools.

Multimedia Appendix 5 General characteristics of studies on the use of digital tools for surveillance.

## Abbreviations

AI: artificial Intelligence
CDC: Centers for Disease Control and Prevention
CHIKV: chikungunya virus
DENV: dengue virus
IoT: internet of things
PPV: positive predictive value
PRISMA: Preferred Reporting Items for Systematic Reviews and Meta-Analyses
ZIKV: zika virus

## Data Availability

The data sets used during the study are available from the corresponding author upon request.
