# Supplementary material for: Use of Digital Tools in Arbovirus Surveillance: Scoping Review
Source: J Med Internet Res. 2024 Nov 18;26:e57476. doi: 10.2196/57476 (PMC11612576; doi:10.2196/57476)

Table S1. The JBI PRISMA-ScR checklist.

| **SECTION** | **ITEM** | **PRISMA-ScR CHECKLIST ITEM** | **REPORTED**  **ON PAGE** |
| --- | --- | --- | --- |
| **TITLE** | 1 | Identify the report as a scoping review. | 1 |
| **ABSTRACT** |  |  |  |
| Structured summary | 2 | Provide a structured summary that includes (as applicable): background, objectives, eligibility criteria, sources of evidence, charting methods, results, and conclusions that relate to the review  questions and objectives. | 1 |
| **INTRODUCTION** |  |  |  |
| Rationale | 3 | Describe the rationale for the review in the context of what is already known. Explain why the review questions/objectives lend themselves to a scoping  review approach. | 1-2 |
| Objectives | 4 | Provide an explicit statement of the questions and objectives being addressed with reference to their key elements (e.g., population or participants, concepts, and context) or other relevant key elements used to conceptualize the review  questions and/or objectives | 2 |
| **METHODS** |  |  |  |
| Protocol and  registration | 5 | Indicate whether a review protocol exists; state if  and where it can be accessed (e.g., a Web | 2 |
|  |  | address); and if available, provide registration  information, including the registration number. |  |
| Eligibility criteria | 6 | Specify characteristics of the sources of evidence used as eligibility criteria (e.g., years considered, language, and publication status), and provide a  rationale. | 2 |
| Information sources | 7 | Describe all information sources in the search (e.g., databases with dates of coverage and contact with authors to identify additional sources), as well as the date the most recent search was  executed. | 4 |
| Search | 8 | Present the full electronic search strategy for at  least 1 database, including any limits used, such that it could be repeated. | 2  (Multimedia Appendix 2) |
| Selection of sources of evidence | 9 | State the process for selecting sources of evidence (i.e., screening and eligibility) included in the  scoping review. | 2 |
| Data charting process | 10 | Describe the methods of charting data from the included sources of evidence (e.g., calibrated forms or forms that have been tested by the team before their use, and whether data charting was done independently or in duplicate) and any processes for obtaining and confirming data from  investigators. | 2-3 |
| Data items | 11 | List and define all variables for which data were  sought and any assumptions and simplifications made. | 3 |

| Critical appraisal of individual sources of evidence | 12 | If done, provide a rationale for conducting a critical appraisal of included sources of evidence; describe the methods used and how this information was used in any data synthesis (if  appropriate). | 3 |
| --- | --- | --- | --- |
| Synthesis of results | 13 | Describe the methods of handling and  summarizing the data that were charted. | 3 |
| **RESULTS** |  |  |  |
| Selection of sources of evidence | 14 | Give numbers of sources of evidence screened, assessed for eligibility, and included in the review, with reasons for exclusions at each stage, ideally  using a flow diagram. | 3  (Multimedia Appendix 1) |
| Characteristics of sources of evidence | 15 | For each source of evidence, present characteristics for which data were charted and  provide the citations. | 3  (Multimedia Appendix 5) |
| Critical appraisal within sources of  evidence | 16 | If done, present data on critical appraisal of included sources of evidence (see item 12). | 3 |
| Results of individual sources of evidence | 17 | For each included source of evidence, present the  relevant data that were charted that relate to the review questions and objectives. | 3-6 |
| Synthesis of results | 18 | Summarize and/or present the charting results as  they relate to the review questions and objectives. | 5-6 |
| **DISCUSSION** |  |  |  |
| Summary of evidence | 19 | Summarize the main results (including an overview of concepts, themes, and types of  evidence available), link to the review questions | 6-8 |

|  |  | and objectives, and consider the relevance to key  groups. |  |
| --- | --- | --- | --- |
| Limitations | 20 | Discuss the limitations of scoping review process. | 8 |
| Conclusions | 21 | Provide a general interpretation of the results with respect to the review questions and objectives, as  well as potential implications and/or next steps. | 8 |
| **FUNDING** |  |  |  |
| Funding | 22 | Describe sources of funding for the included sources of evidence, as well as sources of funding for the scoping review. Describe the role of the  funders of the scoping review. | No funding |

JBI = Joanna Briggs Institute; PRISMA-ScR = Preferred Reporting Items for Systematic reviews and Meta- Analyses extension for Scoping Reviews.

**Figure S1.** PRISMA (Preferred Reporting Items for Systematic Reviews and Meta-Analyses) flow diagram.


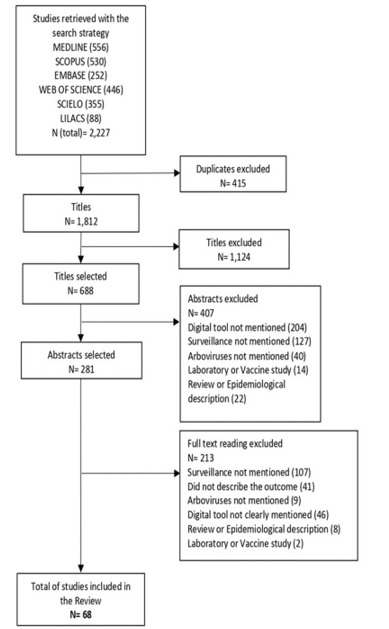

Supplement: Multimedia Appendix 1 [file jmir_v26i1e57476_app1.docx]
