# Supplementary material for: Use of Digital Tools in Arbovirus Surveillance: Scoping Review
Source: J Med Internet Res. 2024 Nov 18;26:e57476. doi: 10.2196/57476 (PMC11612576; doi:10.2196/57476)
Supplement: Multimedia Appendix 2 [file jmir_v26i1e57476_app2.pdf]

Table S2. Search strategy.

Databases	Search equations
PUBMED/ MEDLINE	((((((((((((("arbovirus infections"[MeSH Terms]) OR "dengue"[MeSH Terms]) OR "zika virus"[MeSH Terms]) OR "chikungunya fever"[MeSH Terms]) OR "zika") OR "chikungunya") OR arbovir*) OR "arboviral disease"))) AND ((((("surveillance") OR "public health surveillance"[MeSH Terms]) OR "epidemiological monitoring"[MeSH Terms]) OR "health surveillance") AND ((((((((("technology"[MeSH Terms]) OR "audiovisual aids"[MeSH Terms]) OR "social media"[MeSH Terms]) OR "big data"[MeSH Terms]) OR "mobile applications"[MeSH Terms]) OR "social networking"[MeSH Terms]) OR "innovative tools") OR "new media") OR "games")
SCOPUS	KEY ("arbovirus infection") OR "arboviral disease" OR KEY (dengue) OR KEY ("zika virus") OR KEY ("chikungunya fever") AND KEY ("public health surveillance") OR surveillance OR "health communication" OR "epidemiological monitoring" OR "health information" AND KEY (technology) OR KEY ("audiovisual aids") OR KEY ("social media") OR KEY ("big data") OR "digital tool" OR KEY ("mobile applications") OR "social networking" OR "innovative tools" OR games
EMBASE	('arbovirus'/exp OR arbovirus OR 'arbovirus infection'/exp OR 'arbovirus infection' OR 'dengue'/exp OR dengue OR 'zika fever'/exp OR 'zika fever' OR 'chikungunya'/exp OR chikungunya) AND ('surveillance and monitoring'/exp OR 'surveillance and monitoring' OR 'epidemiological monitoring'/exp OR 'epidemiological monitoring' OR 'medical information'/exp OR 'medical information') AND ('technology'/exp OR technology OR 'audiovisual aid'/exp OR 'audiovisual aid' OR 'social media'/exp OR 'social media' OR 'social network'/exp OR 'social network' OR 'big data'/exp OR 'big data' OR 'game'/exp OR game OR 'mobile application'/exp OR 'mobile application')
WEB OF SCIENCE	TÓPICO: (“arbovirus infection” or "arbovirus" or arbovir* or "dengue" or "zika fever" or "zika virus" or "chikungunya" or "chikungunya fever") AND TÓPICO: ("health public surveillance" or "surveillance" or "health communication" or "health information") AND TÓPICO: ("technology" or "audiovisual aids" or "social media" or "social networking" or "big data" or "mobile applications" or game or innovative tools) Índices: SCI-EXPANDED, SSCI, A&HCI, CPCI-S, CPCI-SSH, ESCI.
BVS/LILACS	(((((("ARBOVIRUS") or "DENGUE") or "DENGUE fever") or "ZIKA virus") or "ZIKA virus infection") or "CHIKUNGUNYA fever") or "CHIKUNGUNYA virus" [Subject descriptor] and ((("PUBLIC HEALTH SURVEILLANCE") or "SURVEILLANCE") or "HEALTH COMMUNICATION") or "HEALTH INFORMATION technologies" [Words] and ((((("TECHNOLOGY") or "AUDIOVISUAL AIDS") or "SOCIAL MEDIA") or "SOCIAL NETWORKING") or "BIG DATA") or "MOBILE APPLICATIONS" or new media or innovative tools or games [Words]
SCIELO	("arbovirus" OR arboviral disease OR "arbovirus infection" or "dengue" or "zika virus" or zika or "chikungunya fever" or chikungunya) AND (surveillance OR "public health surveillance" or information OR health communication) AND ("technology" OR "audiovisual aids" OR "big data" OR "social media" OR "social networking" or new media OR innovative tools OR "mobile applications" or game)
