# Supplementary figures and images for: Use of Digital Tools in Arbovirus Surveillance: Scoping Review
Source: J Med Internet Res. 2024 Nov 18;26:e57476. doi: 10.2196/57476 (PMC11612576; doi:10.2196/57476)

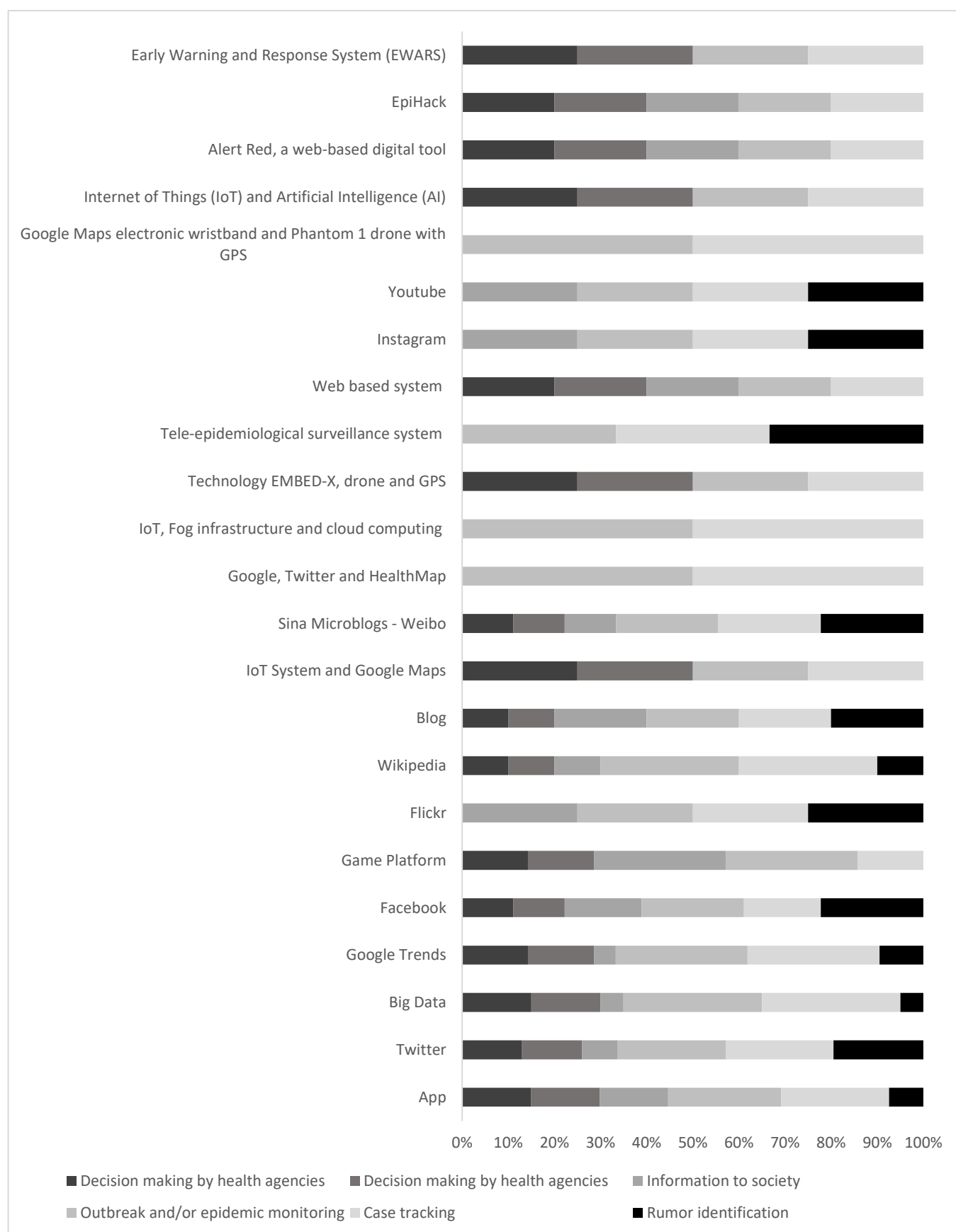

Supplement: Multimedia Appendix 3 [file jmir_v26i1e57476_app3.pdf]

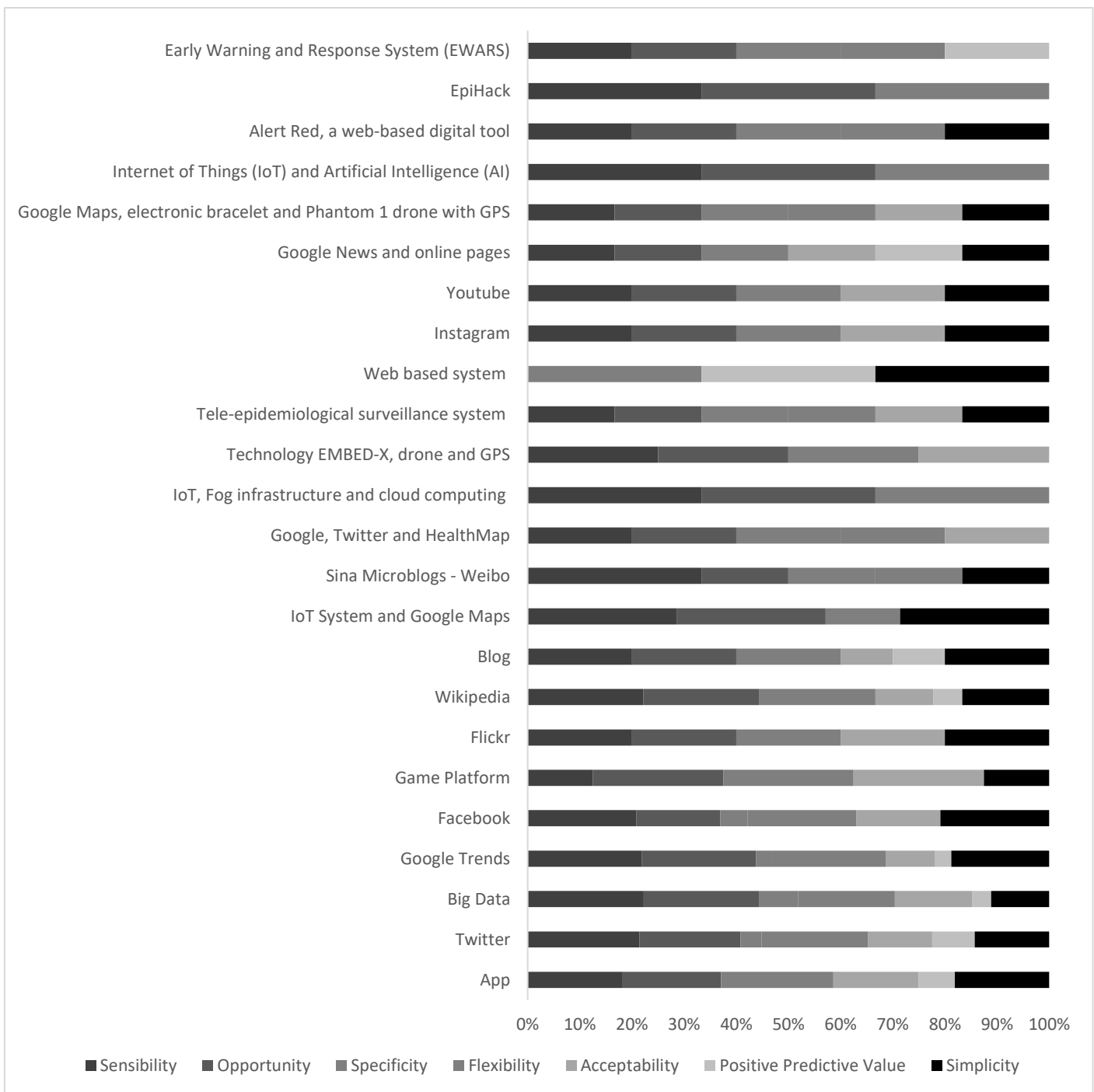

Supplement: Multimedia Appendix 4 [file jmir_v26i1e57476_app4.pdf]
